# Opening new vistas on obsessive-compulsive disorder with the observing response task

**DOI:** 10.3758/s13415-023-01153-w

**Published:** 2024-02-05

**Authors:** Luise Pickenhan, Amy L. Milton

**Affiliations:** Department of Psychology, University of Cambridge, Downing Site, Cambridge, CB2 3EB UK

**Keywords:** Animal models, Obsessive-compulsive disorder, Checking

## Abstract

Obsessive-compulsive disorder (OCD), a highly prevalent and debilitating disorder, is incompletely understood in terms of underpinning behavioural, psychological, and neural mechanisms. This is attributable to high symptomatic heterogeneity; cardinal features comprise obsessions and compulsions, including clinical subcategories. While obsessive and intrusive thoughts are arguably unique to humans, dysfunctional behaviours analogous to those seen in clinical OCD have been examined in nonhuman animals. Genetic, ethological, pharmacological, and neurobehavioural approaches all contribute to understanding the emergence and persistence of compulsive behaviour. One behaviour of particular interest is maladaptive checking, whereby human patients excessively perform checking rituals despite these serving no purpose. Dysfunctional and excessive checking is the most common symptom associated with OCD and can be readily operationalised in rodents. This review considers animal models of OCD, the neural circuitries associated with impairments in habit-based and goal-directed behaviour, and how these may link to the compulsions observed in OCD. We further review the Observing Response Task (ORT), an appetitive instrumental learning procedure that distinguishes between functional and dysfunctional checking, with translational application in humans and rodents. By shedding light on the psychological and neural bases of compulsive-like checking, the ORT has potential to offer translational insights into the underlying mechanisms of OCD, in addition to being a platform for testing psychological and neurochemical treatment approaches.

## Introduction

Obsessive-compulsive disorder (OCD) is a debilitating neuropsychiatric illness with a lifetime prevalence of 3.5%, affecting more 5.4% of women and 1.7% of men (Angst et al., 2004; Fawcett et al., 2020). OCD is characterised by two cardinal symptom clusters: obsessions and compulsions. Obsessions denote unwanted, intrusive, and recurring thoughts, mental images, or impulses that patients struggle to suppress, whereas compulsions describe repetitive and stereotyped behaviours that the individual feels compelled to perform (Van Oppen et al., 1995). Those affected typically have great insight about the irrational nature of their obsessions and subsequent compulsive coping strategies and display self-awareness into the impact their OCD symptoms have on their lives, highlighting the disorder’s often ego-dystonic phenomenology (i.e. obsessive-compulsive symptoms are incongruent with a person’s goals or concept of adaptive behaviour; Clark & O’Connor, 2005; Rasmussen & Eisen, 1992; Rasmussen & Eisen, 1997). In addition to the costs to the individual affected directly by OCD, OCD is estimated to cost UK healthcare providers more than £378 million per year; total societal costs exceed £5 billion annually (Kochar et al., 2023).

Traditionally, OCD has been conceptualised as a disorder in which compulsions are performed to alleviate the distress and anxiety produced by obsessive thoughts (Rachman, 1998; Salkovskis, 1985). Until 2013, when it was reclassified in the fifth edition of the Diagnostic and Statistical Manual of Mental Health Disorders (DSM-V), OCD was considered to be an anxiety disorder, with symptoms reflecting dysfunction of a “security motivation system” (Szechtman and Woody, 2004). This system, it is argued, allows individuals to predict and avoid threats and receive a “safety” or “termination” signal when the threat has been successfully avoided; however, in patients with OCD, this security motivation system fails, either through a loss of the feeling that the threat has been successfully avoided (Szechtman and Woody, 2004) or because the level of threat has been overestimated (Tolin et al., 2006). People with OCD are less tolerant to (Tolin et al., 2003; Rotge et al., 2015) and show increased checking under (Rachman, 2002) conditions of uncertainty, which is usually perceived as anxiogenic. A reduction in anxiety could potentially account for the efficacy of selective serotonin reuptake inhibitors (SSRIs) as first-line treatments for OCD (Völlm et al., 2006; Tang et al., 2016), although as other anxiolytic drugs are not effective (Robbins et al., 2019), SSRIs may instead act to modulate cognitive flexibility (Clarke et al., 2007; Barlow et al., 2015). Furthermore, anxiety and intolerance of uncertainty cannot be the only drivers of OCD. People without OCD also feel increased urges to check if their ongoing levels of perceived threat are elevated (Parrish & Radomsky, 2010, 2011). Intolerance of uncertainty is not specific to OCD, because it is also observed in generalised anxiety disorder (Holaway et al., 2006) and major depressive disorder (Gentes & Ruscio, 2011).

OCD is a heterogeneous disorder, marked by high interindividual variability in how symptoms and core features manifest (Calamari et al., 1999; Mataix-Cols et al., 2005). Several symptomatic clusters have been identified, and more than one can be present in a given person living with OCD. One common cluster revolves around contamination fears (Olatunji et al., 2007; Wahl et al., 2008), whereas another cluster focuses on order and symmetry (Radomsky & Rachman, 2004). Excessive checking is a common and debilitating symptomatic feature of OCD (Strauss et al., 2020), exemplified by individuals feeling compelled to verify repeatedly the completion of mundane tasks without any functional reason for doing so. Excessive checking is amongst the most prevalent symptoms seen in patients with OCD, which often is exacerbated by feelings of anxiety (Sasson et al., 1997; Heyman et al., 2006; Fullana et al., 2010), although not necessarily (Clair et al., 2013). Moreover, perseverative checking is highly representative of the compulsive persistence of behaviour despite aversive consequences for patients’ daily functioning.

The primacy of compulsive behaviour in OCD, rather than the involuntary and intrusive thoughts that characterise obsessions, makes the development of cross-species models of OCD substantially more tractable. These translational models provide the capacity for causal testing of dysfunctional processes and circuits identified in the human patient literature. We review the processes and neural circuits that are dysfunctional in OCD before discussing prominent animal models of OCD that have been used to inform these investigations. Finally, we focus on a relatively new animal behavioural analogue of dysfunctional checking: the Observing Response Task (ORT; Eagle et al., 2014; Morein-Zamir et al., 2018), which we have been using in our lab to characterise the transition from functional to dysfunctional behaviours in OCD.

### Psychological and neural mechanisms of OCD: Evidence from human studies

Understanding the psychological processes that go awry in OCD allows for more targeted understanding of neural circuit differences. These predominantly revolve around working memory dysfunction, memory distrust, excessive error monitoring, impulsivity and deficits in response inhibition, impaired cognitive flexibility, and aberrations in goal-directed learning and habit formation.

## Dysfunctional psychological processes in OCD

One prominent neuropsychological feature compromised in OCD psychopathology is executive functioning, chiefly demonstrated within the context of working memory (Martin et al., 1995) but also in changes in impulsivity and response inhibition and impaired cognitive flexibility.

### Working memory

Multiple studies have observed memory changes in OCD patients. Working memory deficits have been observed in OCD patients across a range of behavioural tests, including the canonical N-back task (Nakao et al., 2009; de Vries et al., 2014; Heinzel et al., 2021), the impaired performance of which was associated with increased activation of frontal brain regions. This included increased activity in the right dorsolateral prefrontal cortex (Nakao et al., 2009) and aberrant frontoparietal activation (de Vries et al., 2014). According to a systematic synthesis of 46 experimental studies conducted by Harkin & Kessler (2011), the working memory deficits associated with compulsive checking in OCD can be attributed to heightened cognitive load.

The reduced working memory capacity of OCD patients led to the proposal of the “memory deficit hypothesis,” whereby OCD patients’ memory abilities were postulated to be inferior to those of healthy individuals (Sher et al., 1983). However, subsequent research has found no significant differences between obsessive-compulsive patients and healthy counterparts in longer-term memory accuracy or vividness of reported details on a memory recall test (Moritz et al., 2009a). This is supported by several meta-analyses (Cuttler & Graf, 2009; Moritz et al., 2009b; Kalenzaga et al., 2020; Dar et al., 2022). These lend their support to the notion that what was initially interpreted as a deficit in memory accuracy was rather a matter of memory *distrust*. Self-assessed memory performance amongst OCD patients can be modulated by their perceived responsibility in a given scenario (Radomsky et al., 2001; Moritz et al., 2007) and is amplified during uncertainty (Toffolo et al., 2016). Furthermore, repeated checking aggravates memory distrust and diminished confidence in OCD patients’ memory-related judgments (van den Hout & Kindt, 2003; Radomsky et al., 2006). Even when environmental certainty was evidently improved, thereby increasing self-reported memory confidence in OCD patients, their actions seldom changed in accordance to enhanced trust in their situational judgment (Vaghi et al., 2017). This illustrates a dissociation between cognitive understanding of contingencies between behaviour and outcome (i.e., patients with OCD know which actions are and are not necessary) versus actual behaviour. This dissociation could drive a feedback cycle of dysfunctional checking, in which checking increases memory distrust, leading to a compulsion to check again.

### Impulsivity and response inhibition

An alternative, not mutually exclusive, view is that patients with OCD show a dissociation in the cognitive understanding of the outcomes and their behaviour but continue to act differently because of impulsivity or deficits in response inhibition. This could be the result of a lack of sensory “completion” signals after performance of an action, leading to repetition of the behaviour until it “feels right” (Frith et al., 2000) or deficits in the inhibition and control of a response once initiated. Deficits in response inhibition have been widely reported in OCD patients (Bannon et al., 2002; Chamberlain et al., 2005). OCD patients show deficits on the Stop-Signal Reaction Time (SSRT) task, a test of action inhibition, compared with their first-degree relatives and unrelated, healthy controls (Menzies et al., 2007). Furthermore, there is a correlation between obsessive-compulsive tendencies and performance on the Go/No-Go task (Abramovitch et al., 2015). A recent meta-analysis (Mar et al., 2022) found consistent slowing of reaction times on the SSRT task in patients with OCD, and when considered in the context of similar impairments (Guo et al., 2018) on the Think/No Think task, which measures the capacity to suppress unwanted thoughts, this suggests a likely deficit in cognitive control that may be shared with other compulsive disorders (Robbins et al., 2019).

### Cognitive flexibility

Cognitive flexibility describes the mental ability to shift attention between different external stimuli or tasks as well as to successfully adapt behaviour in accordance with situational relevancy (Ionescu, 2012; Dajani & Uddin, 2015). Cognitive flexibility therefore denotes a critical executive function that operates under constantly changing environmental demands, alongside working memory and response inhibition (Diamond, 2013). The three mental domains are codependent in that effective attentional control is necessary for cognitive flexibility, which requires inhibition of responses to irrelevant stimuli. OCD patients have been reported to have deficits in cognitive flexibility as measured using the Wisconsin Card Sorting Test (WCST; Boone et al., 1991; Lucey et al., 1997; Okasha et al., 2000; Tükel et al., 2012) and the CANTAB Intra-Extra Dimensional (IED) Set Shifting task (Downes et al., 1989; Sahakian & Owen, 1992). The finding that unaffected first-degree relatives of OCD patients also have impaired IED performance compared with healthy controls (Chamberlain et al., 2007) may suggest that cognitive flexibility is a neuropsychological endophenotype of OCD (Chamberlain et al., 2021; Vaghi, 2021). Crucially, the robustly shown deficits in cognitive flexibility amongst OCD patients, especially those displaying excessive checking, chiefly pertain to fundamental differences in goal-directed and habitual learning processes.

### Goal-directed and habitual learning in OCD

It has been argued (Graybiel & Rauch, 2000) that compulsive behaviours seen in OCD, such as excessive washing or dysfunctional checking rituals, are habits gone awry. This view is supported by studies showing an imbalance between goal-directed and habit-based learning systems in OCD patients (Gillan et al., 2011). While healthy control participants displayed flexible, goal-directed control over their responses by implementing stimulus feedback during learning, OCD patients did not, suggesting an overreliance on habitual responding (Gillan et al., 2014). Additionally, OCD patients have been found to express diminished sensitivity to outcome devaluation (Gottwald et al., 2018), a hallmark test of the goal-directedness of behaviour (Dickinson, 1985). OCD patients also are impaired in adjusting their behaviour in light of contingency degradation, another test of the goal-directedness of behaviour, despite self-reported awareness of reduced causality between actions and outcomes (Vaghi et al., 2019).

Despite some minor variability in the replicability of findings (Cavedini et al., 2010; Rajender et al., 2011), it is widely accepted that goal-based behavioural deficits represent susceptibility markers and endophenotypes of OCD (Dong et al., 2020). Accordingly, the tendency towards aberrant goal-directed learning may be present in asymptomatic relatives of OCD patients without being overtly manifested to the extent of clinical or statistical significance compared with healthy controls. Such an argument finds support from neuroimaging studies, showing task-related functional hypoactivity in the neural correlates of goal-directed behaviour in unaffected relatives of OCD-affected individuals compared to healthy subjects (Vaghi et al., 2017a). Furthermore, these goal-directed performance deficits in OCD patients are associated with reduced functional connectivity between the dorsal lateral prefrontal cortices (dlPFC) and putamen (Vaghi et al., 2017b), and aberrant white matter connectivity in this goal-directed neural circuitry correlates with OCD symptom severity (Vaghi et al., 2017b; Peng et al., 2021).

## Neural correlates and mechanisms of OCD

The wide-ranging nature of psychological dysfunction in OCD is associated with structural and functional differences in cortical and subcortical brain networks in OCD patients.

### Corticostriatal model of OCD

The prevailing neurobiological model of OCD is the corticostriatal account (Pauls et al., 2014; Menzies et al., 2008), supported by multimodal imaging and metabolic alterations in patients compared with healthy individuals (Kwon et al., 2003; Moreira et al., 2017). Accordingly, those corticostriatal impairments also relate to specific domains of neuropsychological deficits, including working memory dysfunction, diminished response inhibition, and cognitive inflexibility (de Vries et al., 2014; Heinzel et al., 2018; Gu et al., 2008).

The neural correlates of OCD pathophysiology include structural grey matter changes (Radua & Mataix-Cols, 2009; Eng et al., 2015) and reductions in resting state functional connectivity in corticostriatal networks (Chen et al., 2016). Moreover, functional connectivity between the putamen and frontal cortical subregions correlates with symptom severity, measured by the Yale-Brown Obsessive-Compulsive Scale (Y-BOCS), in unmedicated OCD patients (Park et al., 2020) and elevated functional connectivity between specific regions within the wider cortico-striatal-thalamo-cortical network, namely the caudate nucleus, orbitofrontal (OFC), anterior cingulate (ACC), and dlPFC (Harrison et al., 2009).

Even in healthy controls, reliance on habits has been associated with shifts in the underlying neural circuitry. However, in OCD there appears to be an imbalance between the neural systems supporting goal-directed and habitual behaviour, and prefrontal control of these. Unmedicated OCD patients show abnormal connectivity between the putamen and caudate nucleus, which negatively correlates with task-switching performance (Peng et al., 2022). Furthermore, there are correlations in genetic risk factors associated with OCD and enlarged striatal structures, such as the nucleus accumbens and putamen (Hibar et al., 2018). There also are differences in connectivity between prefrontal regions, such as the ACC and dlPFC with the caudate (Peng et al., 2021), with monozygotic twins both affected by OCD showing reduced inferior frontal white matter volume compared to dizygotic twin controls (den Braber et al., 2011).

Functional activation differences between OCD patients and healthy controls are most apparent during symptom provocation. Using a personalised symptom provocation procedure, Banca et al. (2015) found deactivation of the circuitries involving the caudate nucleus and prefrontal cortex alongside hyperactivation of the subthalamic nucleus and putamen. Avoidance of, or relief from, the provocation cues produced deactivation of the putamen, leading the authors to speculate that putamen activation is a crucial behavioural modulator. Moreover, they found decreased ventromedial prefrontal (vmPFC) and dlPFC activity as well as reduced caudate activation amongst patients compared with controls, arguing for these anatomical structures’ dysregulation as mechanistic factors giving rise to compulsivity in OCD. This neural activity pattern also involves connections to the ACC amidst related cortical and striatal regions. Thus, the corticostriatal dysfunctions alluded to here are assumed to contribute to cognitive inflexibility by virtue of greater reliance on habitual behaviour (Remijnse et al., 2013; Burguière et al., 2015; Smith & Graybiel, 2022). Notably, this account would explain clinical symptoms, such as excessive checking in OCD as a phenotype and side-effect of impaired cognitive control and pathological habit formation (Goodwin & Sher, 1992; Graybiel & Rauch, 2000; Gu et al., 2008).

While there appear to be common core differences in functional connectivity in patients with OCD, there also are changes in neural function that correlate specifically with different OCD subtypes: imaging OCD patients during symptom provocation produced different functional activation depending on the OCD subtype. While hoarding elicited the greatest activation in the left precentral gyrus and right OFC, washing compulsions correlated with bilateral vmPFC and right caudate nucleus activity, and checking was most strongly associated with neural hyperactivation in striatal regions, such as the putamen and globus pallidus as well as dorsal areas of the prefrontal cortex (Mataix-Cols et al., 2004). Different symptom dimensions being underpinned by distinct neural substrates appears replicable across studies (van den Heuvel et al., 2009; Yu et al., 2022). For instance, Murayama et al. (2013) substantiated the neural differentiation between excessive washing and dysfunctional checking behaviours. They observed, in unmedicated individuals with washing compulsions, hyperactivation in numerous bilateral corticocerebellar brain areas, thus alluding to wider-reaching neural involvement underlying patients’ behavioural pathology. For these washers, insular connectivity may represent an important mechanistic function, supported by its role in disgust and processing aversive stimuli (Ravindran et al., 2020; Straube & Miltner, 2011; Palminteri et al., 2012). In contrast, subjects exhibiting dysfunctional checking demonstrated hypoactivation in the left caudate as well as ACC (Murayama et al., 2013), hence indicating a more specific neural response pattern than their “washing” counterparts. Moreover, ACC activation was significantly correlated with symptomatic severity in compulsively checking patients. This further corroborates a distinct neural correlate as being associated with the symptom dimension of dysfunctional checking compared with an ostensibly more general neural basis for contamination fears and corresponding washing rituals.

As for other mental health disorders, understanding of the mechanisms of OCD can be greatly advanced by the study of translational animal models that are amenable to causal manipulations (Rutherford & Milton, 2022). While obsessions and intrusive thoughts are unlikely to be readily studied in animals (although see Gourley et al., 2021), recent reconceptualisations of OCD (Robbins et al., 2019) that place emphasis on the primacy of compulsions, rather than obsessions, suggest that animal models may be able to provide insight into the disorder.

#### Animal models of OCD

Despite some scepticism (Nestler & Hyman, 2010), animal models are critical for causal studies relating to the neurobiological bases of behaviour (Rutherford & Milton 2022; Vanderschuren et al., 2023). Whereas obsessions remain a challenge to model in animals (Gourley et al., 2021), animal models are highly useful for granting insights into fundamental processes that go awry in mental health disorders, including the recruitment of habit-based and goal-directed learning systems relevant to OCD. However, the validity of a given animal model of human neuropsychiatric illness can only be ascertained by rigorous testing and back-translation (Venniro et al., 2020; Rutherford & Milton, 2022), which can be particularly difficult for disorders in which few effective treatments are available for human patients (e.g. drug addiction; Rutherford & Milton, 2022). Where such treatments are available, animal models can be assessed in terms of their predictive, as well as face and construct validity (Willner, 1986; Geyer & Markou, 1995) and also in terms of their reproducibility, reliability, and robustness of findings.

Animal models of OCD can be broadly defined into ethological models, in which naturalistic behaviour appears compulsive under pharmacological, circuit-based (e.g. optogenetic) or genetic manipulations, and neurobehavioural models, in which compulsive-like behaviour is produced by training animals on specific behavioural tasks. These are then usually validated through the use of pharmacological or neural manipulations that would be predicted to exacerbate or ameliorate OCD-like symptoms in the model.

## Ethological models of OCD

The modelling in animals of complex mental health conditions, such as OCD, will likely benefit from multiple, converging approaches, which would include both laboratory-based studies of behaviour and more ethological approaches (Rutherford & Milton, 2022). Accordingly, the existence of repetitive, seemingly disordered behaviours has been reported in various nonhuman animal species, with a strong association with a stress-inducing contexts. These include canine tail-chasing (Brown et al., 1987), acral licking dermatitis and fur chewing (Rapoport et al., 1992), excessive grooming in mice (Garner et al., 2004), and psychogenic alopecia in cats (Swanepoel et al., 1998). Notably, the association between some of the aforementioned behaviours and stress alludes to the correlation and comorbidity between obsessive-compulsive disorder or clinical anxiety (Adams et al., 2018). As these symptoms only occur in a small portion of the larger population, they may allow for the study of mechanisms instigating OCD-related pathology, including polymorphic susceptibility and epigenetic factors (d’Angelo et al., 2014). It may hence be argued that the above-described animal behaviour in response to external stressors illustrates something akin to human compulsions, the latter often reported to serve the purpose of alleviating emotional discomfort, alongside pathological habit formation. However, despite their face validity, it is not clear whether these aberrant responses reflect naturally occurring coping mechanisms to anxiety, rather than modelling the compulsive behaviour observed in OCD.

## Genetic models of compulsive-like behaviour

Given a genetic basis for the pathogenesis of obsessive-compulsive disorder, implicating serotonergic and glutamatergic polymorphisms amongst other factors (Walitza et al., 2010; de Salles Andrade et al., 2019; Walitza et al., 2012), several genetic mouse models with OCD-like, usually reliant on ethological measures, have been developed. However, these models do not target the same mutations thought to play a role in the human condition, but rather instigate genetic modifications to produce behaviours resembling compulsive symptoms in humans. Doing so yields good face validity because of the readily observable behavioural component of OCD. For example, dopamine transporter knock-down (DAT KD) mice, which show a 70% increase in extracellular dopamine levels (Zhuang et al., 2001), show behavioural aberrations, including hyperactivity, augmented reward sensitivity (Pecina et al., 2003), and repetitive grooming behaviour, ostensibly analogous to perseverative and compulsive rituals performed by human OCD patients (Berridge et al., 2005). Similar behaviours have been observed in DAT knock-out rats (Reinwald et al., 2022).

One of the most well-established genetic models of OCD is the knockout of the *SAPAP-3* gene, which produces a protein that facilitates glutamatergic transmission (Züchner et al., 2009). *SAPAP-3* knockout mice display excessive grooming (Welch et al., 2007; Ting & Feng, 2011), which can be ameliorated by administering the selective serotonin reuptake inhibitor fluoxetine (Welch et al., 2007; Manning et al., 2021). Such findings support the *SAPAP-3* knockout model’s predictive validity. *SAPAP-3* knockout mice also exhibit aberrant learning, with an overreliance on habitual learning and propensity to aberrant habit formation (Hadjas et al., 2019; Ehmer et al., 2020a). Furthermore, *SAPAP-3* knockout mice show compulsive-like behaviour on the signal attenuation task (Ehmer et al., 2020b) and deficits in behavioural flexibility (van den Boom et al., 2019). At the neural level, *SAPAP-3* knockout mice have abnormal serotonergic and dopaminergic signalling in the lateral and medial OFC and the striatum (Welch et al. 2007; Wood et al., 2018; Lei et al., 2019). They also show selective deficits in signalling between corticostriatal synapses without affecting thalamostriatal synaptic activity (Wan et al., 2014).

However, it should be noted that the *SAPAP-3* knockout genotype is also characterised by elevated anxiety (Welch et al., 2007), which, given its robust link to OCD, may affect animals’ task performance where stress can play a modulatory, anxiety-inducing role with possible consequences for compulsive behaviours. Indeed, it has recently been argued that *SAPAP-3* knockout mice may more accurately reflect repetitive behaviours of relevance to Tourette’s syndrome and trichotillomania, not only OCD (Lamothe et al., 2023; Schreiweis & Burguière, 2022). This is partly corroborated by Bienvenu et al. (2009) who found an association between polymorphisms in the human version of the *SAPAP-3* gene and behavioural pathologies, including body-focused compulsive disorders, such as trichotillomania, but no relationship between *SAPAP*-related genetic variation and OCD.

## Neurobehavioural models of OCD

Neurobehavioural models of OCD have been used to induce compulsive-like behaviour by targeting a variety of psychological processes thought to contribute to OCD in humans. These models are usually validated through pharmacological or neural manipulations that would be predicted to enhance or reduce OCD-like behaviour.

### Signal attenuation

The signal attenuation procedure arises from the perspective that compulsive behaviour stems from deficient feedback in relation to the performance of goal-directed responding (Joel & Avisar, 2001; Joel, 2006a; 2006b). Under “normal” conditions, feedback would prevent further responding once the goal had been acquired, but under conditions of reduced feedback signals, behaviour continues in a dysfunctional and eventually compulsive manner (Goltseker et al., 2015). Using the “signal attenuation task,” Joel and colleagues have shown that perseverative lever pressing can be reduced by acutely administering SSRIs, such as fluvoxamine and paroxetine (Joel et al., 2004), giving the model good predictive validity. Furthermore, because of its strong theoretical framework, the signal attenuation model offers good construct validity. However, it is limited by the inability to test for the behavioural effects of repeated or chronic drug administration as a result of their influence on behavioural acquisition during early stages of the procedure (Alonso et al., 2015).

### Reversal learning and behavioural flexibility

The cognitive flexibility deficits reported in OCD (Chamberlain et al., 2008) have been modelled in animals by using behavioural tasks, such as reversal learning. Deficits in reversal learning have been reported for *SAPAP-3* knockout mice (van den Boom et al., 2019) and OFC-lesioned monkeys (Dias et al., 1996), rats (Boulougouris et al., 2007), and monkeys with selective 5-HT depletion in the OFC (Clarke et al., 2004, 2008). Recently, Hatakama et al. (2022) have demonstrated that SSRI administration augmented behavioural rigidity during reversal learning in mice, by down-regulating *5-HT*_*2C*_ receptor signalling in the OFC. Conversely, measuring neurochemical signalling in the marmoset caudate nucleus has shown reversal learning to be modulated by dopaminergic activity (Clarke et al., 2011). Importantly, these findings collectively implicate two major neurotransmitter systems associated with clinical OCD, serotonin and dopamine, in nonhuman analogues of OCD-related behavioural impairments. Additionally, they substantiate the mechanistic role of both cortical and striatal substrates innervated by these neuromodulator systems in aberrations of behavioural flexibility, as demonstrated by reversal learning. Thus, the procedure’s validity for modelling compulsive-like behaviour in animals needs further corroboration in terms of yielding remedial effects of pharmacological agent administration. Targeting serotonin and dopamine activity, as well as glutamate, may grant novel insights into their specific neurochemical function within pathological manifestations of behavioural flexibility across species.

### Exaggerated habit learning

Aberrant habit formation is considered a core feature of the behavioural pathogenesis of compulsions in human OCD patients (Graybiel & Rauch, 2000; Gillan et al., 2016). Much has been learned about the neural basis of habit learning from studies in animals. Corbit & Janak (2010) have shown that both instrumental and pavlovian learning require the dorsomedial striatum (DMS), the homologue of the human caudate nucleus, while the dorsolateral striatum (equivalent to the putamen) underlies stimulus-response learning (Yin & Knowlton, 2006). Furthermore, the transition from goal-directed to habitual behaviour is associated with a shift in the requirement for both striatal (Vanderschuren & Everitt, 2004; Belin & Everitt, 2008; Zapata et al., 2010) and amygdala subnuclei (Murray et al., 2015). The modulation of goal-directed action is mediated by dissociable structures within the rodent prefrontal cortex, with lesions of the prelimbic cortex resulting in insensitivity to changes in goal value regardless of training duration (Killcross & Coutureau, 2003) and lesions of the infralimbic cortex augmenting sensitivity to reward value, independent of limited or extended training exposure (Coutureau & Killcross, 2003).

Ultimately, animal models of psychiatric illnesses, including obsessive-compulsive disorder, are constrained by the currently incomplete understanding of the full complexity and heterogeneity seen in their human analogues. This is pertinent for OCD, given its multiple symptomatic dimensions, thought to be underpinned by distinct neural correlates. Therefore, it appears justified to focus on a specific subtype within the wider obsessive-compulsive symptomatic spectrum for neurobehavioural modelling in animals, ideally one that aptly relates to different neuropsychological deficits associated with OCD. Substantiated by its diagnostic predictiveness for OCD (Stasik et al., 2012), this fosters the case for translationally operationalising dysfunctional checking to elucidate the neurobehavioural mechanisms of habit-based, compulsive-like pathology in OCD across species.

## Pharmacological models of OCD

The most widely used pharmacological model of OCD has targeted dopaminergic signalling through the subchronic administration of the D_2/3_ dopamine receptor antagonist quinpirole. This has been shown to induce repetitive, compulsive-like checking on an open-field task (Szechtman et al., 1998, 2001), inducing bouts of checking that resembles compulsive checking in OCD (Dvorkin et al., 2006). Quinpirole also modulated behavioural flexibility in marmosets when infused directly into the striatum (Horst et al., 2019). However, it should be noted that although subchronic quinpirole increased checking on the Observing Response Task (Eagle et al., 2014, 2020), the effects were strongest on functional rather than dysfunctional checking, and it also reduced the capacity of treated rats to discriminate between rewarded and unrewarded levers when their identities were unsignalled (see below). Considering the link between uncertainty and information-seeking (Anselme et al., 2013), it may be the case that quinpirole increases checking due to more generalised effects on uncertainty (Eagle et al., 2020). This may provide an account for the finding that administering dopamine receptor antagonists, such as haloperidol, does not produce reductions in compulsive-like behaviour (de Carolis et al., 2011).

### The observing response task as a novel model of OCD

It is becoming increasingly recognised that there is prominent symptomatic heterogeneity between patients with mental health conditions and that symptoms can overlap between disorders; recognition of these facts has given support to the increasingly prominent transdiagnostic approach of dimensional psychiatry (Cuthbert & Insel, 2013; Nusslock & Alloy, 2017). However, the focus on heterogeneity does not negate the value of selecting a specific subtype of OCD and operationalising it to acquire a better grasp of prevailing behavioural and neural mechanisms within OCD.

An example of this approach is the use of the ORT to study compulsive-like checking (Eagle et al., 2014; Morein-Zamir et al., 2018), which has several advantages. One is that excessive checking is amongst the most prevalent symptoms seen in obsessive-compulsive patients, often exacerbated by feelings of anxiety (Sasson et al., 1997; Heyman et al., 2006; Fullana et al., 2010), although not necessarily so (Clair et al., 2013). Moreover, perseverative checking is highly characteristic of the compulsive persistence of behaviour despite aversive consequences for patients’ daily functioning. While checking can be adaptive (and arguably necessary to reduce uncertainty and help to make informed decisions), when its performance becomes excessive and devoid of yielding any further information that would facilitate decision-making, the behaviour enters dysfunctional and, as seen in OCD, pathological territory (Strauss et al., 2021; Wake et al., 2022). Dysfunctional checking compulsions not only represent a prevalent symptom dimension but are the only significant predictor of receiving a diagnosis of OCD (Stasik et al., 2012).

Second, excessive checking behaviour is relevant to the array of neuropsychological impairments observed in OCD, including memory distrust, heightened cognitive load with resulting deficits in working memory function, as well as excessive performance monitoring and error-related negativity (Nakao et al., 2007; Harkin & Kessler, 2011; Heinzel et al., 2018; Weinberg et al., 2015). Furthermore, persistent checking despite no rational reason to execute this illustrates the behavioural pathology underlying cognitive rigidity and, pertinently, aberrant goal-directed learning and habit formation in OCD.

**Fig. 1:**
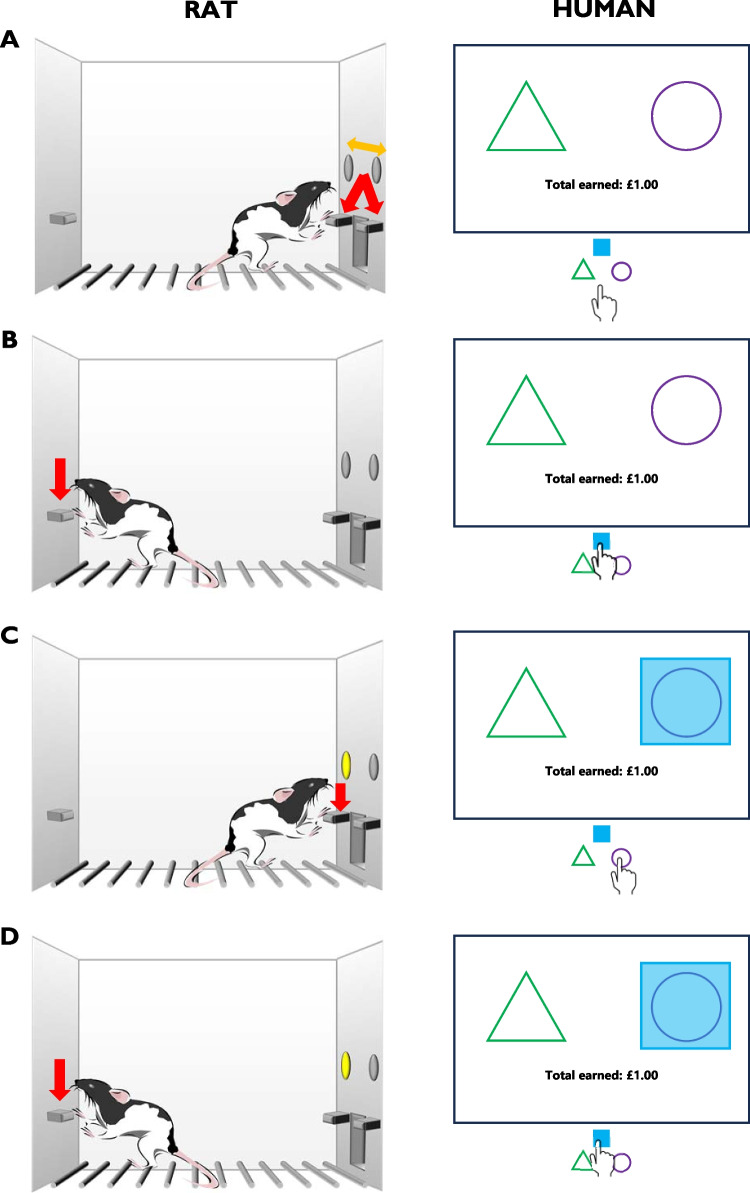
Observing Response Task for rats (left) and humans (right). The task is conducted in operant chambers for rats and on a computer screen with a keyboard for humans. (**A**) Basic task schedule. Two response manipulanda are presented, and pressing (red arrows/pointing) on one of these is deemed a correct response and rewarded with a sucrose pellet for the rats, and the presentation of a pleasant noise and points for the humans. The correct response changes throughout the session (yellow arrow). (**B**) The currently correct response can be indicated through making a functional checking response. For the rats, the checking lever is presented on the back of the chamber. For the humans, a checking key is available. (**C**) Pressing the observing lever illuminates a cue light above the currently correct lever for the rats, and the presentation of a light blue cue square behind the currently correct symbol for the humans. As these responses provide information, they are deemed “functional” observing responses. Responding on the correct manipulandum will be rewarded for as long as that response is correct, regardless of whether the cue is still presented. (**D**) Pressing of the observing lever or key while the cue is still present provides no information and is deemed a “dysfunctional” observing response.

Third, excessive checking is readily studied in animal models. The operationalisation of dysfunctional checking in animals was pioneered by Szechtman et al. (1998), having modelled the compulsive-like behaviour in rats by administering quinpirole. Doing so subsequently turned their normal open-field checking into its perseverative analogue, akin to a shift from adaptive, functional checking for information acquisition’s sake to the persistent, pathological symptom in OCD. Interestingly, quinpirole-induced compulsive-like checking in rats has been reported to be suppressed by administration of the nonselective serotonin *5-HT*_*1A/2A/2B/2C*_ receptor agonist m-chlorophenylpiperazine (Tucci et al., 2013, 2015). Complementing the more ethological approach of the Szechtman group, we have focused on using an operant task for assessing dysfunctional checking: the Observing Response Task (ORT; Eagle et al., 2014). This fully translational task was developed in parallel to a human analogue task (Morein-Zamir et al., 2018). Briefly, in the rodent version, rats learn to respond on one of two levers to receive reinforcement, with the correct lever changing unpredictably throughout the session. Rats can identify the currently correct lever by pressing a third, “observing” lever, illuminating a light cue over the correct lever. These functional Observing Lever Presses (OLPs) can be distinguished from dysfunctional Extra Observing Lever Presses (eOLPs)—the latter having no programmed consequences (Fig. 1). Although it is possible to earn reinforcers without using the checking lever, most individuals will use it to guide behaviour, particularly under conditions where the reinforcement contingencies and the switching of the correct and incorrect levers are made more uncertain (Eagle et al., 2020; Vousden et al., 2020) or the consequences of an incorrect response are made aversive (Vousden et al., 2020). Under baseline conditions, both rodents and humans will typically check at least once every 1–2 minutes on task, with rates increasing under the challenge conditions of uncertainty or punishment of incorrect checking (Eagle et al., 2020; Vousden et al., 2020; Morein-Zamir et al., 2018), although these averages do not reflect the profound individual variability in checking responses. Furthermore, the ORT distinguishes between functional and dysfunctional checking shown by the same individuals, in both rodents and humans, and can be readily used to assess interindividual variability in checking (Eagle et al., 2020; Vousden et al., 2020).

To date, the literature on the ORT is relatively small (Table 1), but investigations have been conducted into both the neural basis of the task and individual differences in task performance. D’Angelo et al. (2017) examined the effects of lesioning brain regions implicated in the pathophysiology of OCD, namely the medial PFC, nucleus accumbens core, and the dorsal striatum, on checking behaviour in rodents as measured by the ORT. Medial PFC lesions selectively augmented functional checking during informative cue presentation, whereas lesions to the nucleus accumbens core resulted in increases in both functional and excessive checking. These findings indicate both structures to be involved in the control of checking behaviour, possibly due to their function in how uncertainty of reinforcement is processed, and arguably promoting excessive and dysfunctional checking analogous to OCD. This interpretation was corroborated by Eagle et al. (2020) who showed that administering the selective D_2/3_ receptor agonist quinpirole in rats increased checking behaviour during uncertainty, though possibly through effects on working memory, as nonspecific task measures (such as discrimination between the correct and incorrect levers in the absence of the cue light) were also impaired by quinpirole administration. Furthermore, an interaction between checking behaviour on the ORT and pavlovian cue reactivity has been repeatedly reported (Eagle et al., 2020; Vousden et al., 2020). Following pavlovian autoshaping procedures, rats can be classified as sign-trackers (approaching and engaging with a cue predictive of reward) or goal-trackers (approaching and engaging with the location of reward delivery). Both behavioural and neural differences have been documented between these two phenotypes (Flagel et al., 2007; Robinson & Flagel, 2009). Whereas sign-trackers show marked increases in dysfunctional checking under conditions of uncertainty, goal-trackers show increased functional checking under conditions of perceived threat (Vousden et al., 2020).

**Table 1:** Key findings obtained from published studies utilising the ORT

Publication	Species	Manipulation	Key findings
Eagle et al. (2014)	Rats	Pharmacological (quinpirole administration)	Following quinpirole administration, uncertainty increased functional and dysfunctional checking behaviour
Eagle et al. (2020)	Rats	Pharmacological (quinpirole administration)	Quinpirole administration in rats classified as sign-trackers increased dysfunctional checking, which was amplified by uncertainty. Goal-tracking animals showed heightened functional checking
D’Angelo et al. (2017)	Rats	Lesions	Lesioning the rodent medial PFC increased functional checking; lesions to the nucleus accumbens increased functional and dysfunctional checking during uncertainty
Vousden et al. (2020)	Rats	Behavioural	Anxiogenic, aversive stimuli increased functional checking without modulating dysfunctional checking. Rats classified as sign-trackers displayed heightened dysfunctional checking amplified by increased uncertainty
Morein-Zamir et al. (2018)	Humans	Behavioural	Punishment led to increased checking in a nonclinical sample, including reduced sensitivity to the aversive consequences of their checking. A clinical sample diagnosed with OCD displayed higher baseline checking as well as markedly greater insensitivity to punishment compared with controls

Considering the heterogeneity in the levels of checking in patients with OCD, another advantage of the ORT as a model is the degree to which individual differences in checking have been observed. These have been assessed both by pharmacological manipulations and by investigating naturally occurring individual differences. One potential source of individual variability in checking relates to sensitivity to reward-related cues. Sign-tracking, indicated by reward cue-driven attentional capture (“incentive salience”), has been implicated in greater severity of addictive and obsessive-compulsive behaviours in humans (Albertella et al., 2019; Watson et al., 2019). Sign-tracking rats have also been observed to show higher levels of dysfunctional checking on the ORT (Eagle et al., 2020; Vousden et al., 2020). Furthermore, the behavioural phenotypes of sign-tracking and goal-tracking are well-documented as being associated with drug-seeking in the addiction literature (Flagel et al., 2009; Kucinski et al., 2018). This is illustrated by observations of the former’s heightened susceptibility to cue-driven reward-seeking rather than goal-directed behaviour based on action-outcome associations, as associated with goal-tracking (Colaizzi et al., 2020). Moreover, testing sign-trackers and goal-trackers on an aversive version of the ORT (aORT), in which incorrect responding was punished with electric footshocks, showed general increases in functional checking throughout the population and some increases in dysfunctional checking at greater shock magnitudes (Vousden et al., 2020). Crucially, whereas goal-trackers and intermediate animals performed fewer dysfunctional eOLPs once the shock contingency was removed, sign-trackers did not decrease their dysfunctional checking behaviour after the contingency between maladaptive checking and shock delivery had been decoupled. This aligns with the notion of persistent, dysfunctional behavioural aberrations analogous to compulsive checking in humans, as well as pointing to the phenotypic susceptibility of sign-tracking subjects to engage in compulsive behaviour, as described in the addiction literature (Flagel et al., 2009; Tomie et al., 2008; Robinson et al., 2018; Schettino et al., 2022). This emerging research hence supports the utility of the ORT as an objective translational tool for illuminating underlying processes akin to the maladaptive checking observed in OCD patients, allowing complementary studies to be conducted in rodents (which would allow for causal manipulations of checking through pharmacological and neural manipulations) and humans (both healthy participants and patients, where there is potential for functional imaging and subjective, in addition to objective, measures).

### Conclusions and future directions

OCD is a highly heterogenous disorder, associated with a number of cognitive and neurobiological differences between OCD patients and neurotypical populations. Considering the heterogeneity of the disorder and the value of convergent approaches (Rutherford & Milton, 2022), different animal models have been developed to study specific processes and mechanisms underlying OCD.

A relatively new addition to these approaches is the ORT, which was developed to be translational between rats and humans and to distinguish objectively between functional and dysfunctional versions of the same checking behaviour. The ORT has the advantages of measuring wide-ranging individual differences in checking, in addition to a rich set of nonchecking measures that allow for the assessment of other psychological processes that may go awry in OCD. For example, it is possible to measure memory performance as determined by the capacity of animals to remember which lever is currently correct, and inhibitory control via the suppression of incorrect responses on the ongoing task. The development of variants of the ORT, including the aversive ORT (in which incorrect responses are punished), probe tests in which the contingency between checking and information is degraded, and a version in which checking is directly punished provide the possibility of measuring the impact of aversive outcomes, reliance on habits, and the compulsive nature of checking as measured by resistance of checking to punishment.

To date, alterations in behaviour on the ORT have been demonstrated with lesions to key regions in the neural circuits underlying OCD, pharmacological manipulations targeting key neurochemical changes in OCD (and validated in other animal models), and individual differences in reward cue reactivity that have been associated with compulsive behaviour in addiction. We argue that the ORT is a valuable addition to the animal analogues of OCD, although we acknowledge that a key focus of future work will be to determine whether dysfunctional checking is habitual (e.g. with contingency degradation procedures) and compulsive (e.g. by directly punishing checking). These behavioural questions would ideally be addressed alongside investigations into the circuit-level mechanisms that underpin the transition from adaptive to maladaptive checking behaviour in rodents and humans. Furthermore, given the sex differences reported for the symptomatic manifestation of human OCD (Raines et al., 2018) and female animals being historically underrepresented in biobehavioural research (Shansky, 2019), testing for possible sex differences in ORT performance, and (dys)functional checking behaviour specifically, may be of interest to allow more nuanced and robust conclusions.

Ultimately, converging and back-translational approaches across a range of genetic, pharmacological, and neurobehavioural models should combine to not only provide new vistas on OCD but also to inform the identification of new treatment targets for this highly debilitating disorder.
