# Correction: Opening new vistas on obsessive-compulsive disorder with the observing response task

**DOI:** 10.3758/s13415-024-01187-8

**Published:** 2024-04-17

**Authors:** Luise Pickenhan, Amy L. Milton

**Affiliations:** Department of Psychology, University of Cambridge, Downing Site, Cambridge, CB2 3EB UK

**Correction: Cogn Affect Behav Neurosci**

10.3758/s13415-023-01153-w

The original article has been updated to correct the article title from “Special issue: preclinical animal models and assays of neuropsychiatric disorders: old problems and new vistas” to “Opening new vistas on obsessive–compulsive disorder with the observing response task”.

